# Family resemblance in color‐patch size is not affected by stress experience in a cichlid fish

**DOI:** 10.1002/ece3.70009

**Published:** 2024-07-21

**Authors:** Angelika Ziegelbecker, Kristina M. Sefc

**Affiliations:** ^1^ Institute of Biology University of Graz Graz Austria

**Keywords:** animal model, carotenoid coloration, cichlid fish, color patch size, color pattern, condition‐dependent signaling, heritability

## Abstract

Animal body coloration is often linked to social dominance and mating success. This is because it can carry information on an animal's body condition and competitive ability by reflecting the genetic quality of individuals or by responding to their current or past living conditions. The present study investigates genetic and environmental effects on a conspicuous color pattern of the cichlid fish *Tropheus* sp. black “Ikola,” in which the size of a carotenoid‐based yellow area on the body co‐varies with social dominance. To examine environmental plasticity of the color pattern, we tested for effects of early‐life stress, induced by reduced feeding of juveniles prior to color pattern formation, as well as effects of a stress treatment administered to fully colored adult fish. None of the stress treatments affected the color pattern as quantified by the width of the yellow bar. However, offspring bar width was correlated to parental values in mid‐parent‐mid‐offspring regression analyses, and animal models estimated significant additive genetic effects on bar width, indicating heritability of the trait. Depending on the random effects structure of the animal models (i.e., whether including or excluding maternal and brood effects), narrow‐sense heritability estimates for bar width ranged between 0.2 and 0.8, with the strongest statistical support for the highest estimate. In each of the alternative models, a large proportion of the total variance in bar width was explained by the included random effects, suggesting that bar width is strongly determined by genetic factors or shared maternal and brood environments, with limited scope for environmental influences later in life.

## Graphical abstract

We investigated inheritance and environmental effects on a carotenoid‐based color pattern linked to social dominance in a cichlid fish (*Tropheus* sp.). The investigated trait was insensitive to experimental stress treatments during juvenile development as well as during adulthood, while trait value correlations among relatives demonstrated heritable and maternal effects on color pattern variation.

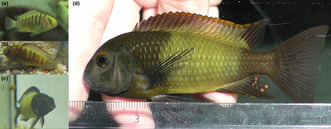

## 1 INTRODUCTION

Animal color patterns are not only among the most striking, but also among the most variable manifestations of biological diversity. While discrete color pattern differences such as between species or color morphs are typically genetically controlled (e.g., O'Quin et al., 2012), the continuous variation in color patch size, chroma, and other elements of color patterns that is observed within species is to various degrees influenced by both genetic and environmental factors (Brooks & Endler, 2001; Fuller & Travis, 2004; Griffith et al., 2006; Lee et al., 2018; Lewandowski & Boughman, 2008; Tibbetts, 2010). Reflecting these influences, intraspecific color pattern variation can carry information about individual differences in quality and condition and hence provide cues for mate choice and competitive interactions (Griffith et al., 2006; Hernandez et al., 2021; Weaver et al., 2017). In order to reflect individual quality, color pattern traits may be linked to intrinsic physiological conditions or may be regulated via punishment of cheaters (Bachmann et al., 2017; Biernaskie et al., 2014; Culbert & Balshine, 2019; Higham, 2013). In case of heritable color pattern variation, links to individual quality can arise from genetic correlations between color pattern formation and behavioral or physiological traits (Roulin, 2016; Sly et al., 2022). On the other hand, environmental sensitivity of color patterns includes responses to social stimulation (Dey et al., 2015; Dijkstra et al., 2007), immunological challenges (Houde & Torio, 1992; Mougeot et al., 2010), and nutrition (Grether et al., 1999). Environmentally plastic body coloration can therefore relate to body condition in “real‐time,” but in addition to reflecting current condition, body color may also indicate past conditions dating back to the previous molt of an adult bird (DePinto & McGraw, 2022) or to various stages of its ontogenetic development (DeKogel & Prijs, 1996; Hubbard et al., 2015; Naguib & Nemitz, 2007; Wilson et al., 2019). Since diverse physiological and cognitive properties can likewise covary with conditions experienced during early development (Metcalfe & Monaghan, 2001; Monaghan, 2008), early life‐induced variation in body color is a potentially relevant cue in mate choice and competitive interactions later in life (Naguib & Nemitz, 2007; Royle et al., 2005).

Given its dependency on dietary uptake and potential allocation trade‐offs between ornamentation and vital functions, carotenoid pigment‐based coloration has traditionally been associated with honest signaling of physiological conditions (Griffith et al., 2006; Svensson & Wong, 2011). Color properties of carotenoid‐based ornaments are controlled by carotenoid uptake, modification, and deposition in the integument and opposing processes such as mobilization of integumentary carotenoids for other purposes or break‐down into uncolored derivatives (Svensson & Wong, 2011). These metabolic processes allow carotenoid‐based coloration to respond to environmental and physiological changes (e.g., Rosenthal et al., 2012). Attention has also been drawn to the condition dependence and the resulting information content of melanin‐based coloration (Griffith et al., 2006; Hegyi et al., 2020; Minias et al., 2019; Roulin, 2016; Sly et al., 2022). Melanin pigments are endogenously synthesized from amino acids and observed correlations between integumentary melanin concentration (perceived as blackness) and body condition have been hypothesized to arise from production or display costs or from pleiotropic genetic effects (Hegyi et al., 2020; Roulin, 2016). For both melanin‐ and carotenoid‐based coloration, evidence for environmental effects on chroma and brightness and low heritability estimates of these traits support the potential for condition‐dependent, plastic signaling (Evans & Sheldon, 2012; Hubbard et al., 2015; Quesada & Senar, 2009).

Not only color properties such as darkness or chroma but also the size of colored patches has been linked to body condition (Dijkstra et al., 2007; Minias et al., 2019; Naguib & Nemitz, 2007; Sly et al., 2022; Walker et al., 2013). In fish, amphibians and reptiles, areas of color patches are defined by the distributions of chromatophores in the integument. As demonstrated in zebrafish, the spatial organization of chromatophores during pattern formation is controlled by interactions between the pigment cells (Patterson & Parichy, 2019; Watanabe & Kondo, 2015). Once color pattern formation has been completed, plastic responses of color patch size to fluctuations in body condition may seem unlikely. However, in contexts such as sexual maturation, sex reversal, or adaptation to novel environments, color patterns can change within few weeks (Leclercq et al., 2010; Sköld et al., 2015; Warner & Swearer, 1991). Particularly in fish, body coloration can also be quickly adjusted via physiological color changes, that is, movements of pigment organelles within chromatophores (Sköld et al., 2013). Moreover, even if the area of a color patch was irreversibly fixed in the process of color pattern formation, its size may depend on the conditions experienced during or before that period and preserve this information into adulthood. Early life effects on adult color patch size have been demonstrated mostly in birds (e.g., Jensen et al., 2006; Walker et al., 2013; Wilson et al., 2019) and also exist in fish (Ruell et al., 2013). Other studies, in contrast, revealed high heritability of color patch size, suggesting that individual variation was mostly due to additive genetic effects (Brooks & Endler, 2001; Houde, 1992; Ng et al., 2013).

**FIGURE 1:**
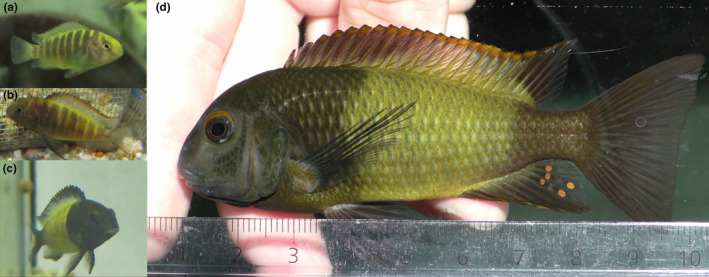
*T*. “Ikola” color pattern formation. (a) juvenile aged approx. 2 months, (b) juvenile aged approx. 7 months, (c) young adult at age 8 months, showing adult color pattern during a social interaction, (d) adult at age 2.2 years. In (d), the fish shows subdued color intensity as a response to being handled.

In fish, numerous species of the large and diverse family of Cichlidae rely on color patterns and color properties for species and mate recognition, mate choice, and social interactions (Maan & Sefc, 2013). Color patterns of cichlid fishes are typically based on melanin, carotenoids, and structural colors, while pteridine pigments have only been detected in a few species (Maan & Sefc, 2013; Santos et al., 2014; Sefc et al., 2014). In the current study, we examine potential mechanisms influencing the variation in a conspicuous color pattern, a wide yellow bar displayed on the flank of an otherwise black body, which characterizes adults of a color variant of the genus *Tropheus*, *Tropheus* sp. black “Ikola” (hereafter *T*. “Ikola”), from Lake Tanganyika, Africa. The color pattern is sexually monomorphic and forms during sexual maturation (Figure 1). Among wild‐caught individuals, the width of the yellow bar (“relative bar width” RBW, quantified relative to body length) varies from 27 to 40% (*n* = 140 individuals; Ziegelbecker & Sefc, unpublished). Melanophore density is lower in the yellow bar area than in the black skin regions (Ziegelbecker et al., 2018). Xanthophores are present in both yellow and black‐colored skin regions, but the yellow‐colored skin contains a three times higher concentration of carotenoids per cm^2^ skin area than the black‐colored skin. The difference in integumentary carotenoid content between wide‐barred and narrow‐barred fish of the same size can amount to up to 25 μg (Ziegelbecker et al., 2021). The coloration of wide bars is therefore associated with elevated carotenoid allocation compared to narrow bars of the same color intensity. Nonetheless, it is an intriguing aspect of this color pattern that bar width can be interpreted in the context of carotenoid signaling as well as in the context of melanin signaling: the larger the area of the yellow bar, the smaller the area displaying the black melanin. While social dominance can be related to either melanin or carotenoid patch size (Griffith et al., 2006), previous experiments with the yellow‐barred *T*. “Ikola” revealed a positive correlation between RBW and success in female–female contest competition (Ziegelbecker et al., 2018). Furthermore, RBW was negatively correlated with the latency of intrusion into unfamiliar tank areas as well as with aggression elicited in male rivals (Ziegelbecker et al., 2021). This raises the question, which factors—genetic and/or environmental—contribute to variation in RBW and the potential signaling function of the trait. To examine environmental plasticity of the color pattern, we used a full‐sib split brood rearing experiment to test for effects of early‐life stress, induced by reduced feeding of juveniles, on the bar width expressed in the adult pattern. Furthermore, to test whether bar width responds to stress experienced *after* the formation of the adult color pattern, we temporarily exposed adult fish to adverse conditions induced by a combination of fasting, social deprivation, unstructured tank environment, and repeated chasing. Finally, we examined genetic and environmental components of variation in bar width using parent–offspring regression analyses and animal models.

## 2 MATERIALS AND METHODS

Housing and handling of the fish was covered by permit number BMWFW‐66.007/0004‐WF/V/3b/2016 issued by the Federal Ministry of Science, Research and Economy of Austria. The experiments were carried out with the approval of the ethics committee of the University of Graz (permit numbers GZ. 39/45/63 ex 2019/20 and GZ. 39/95/63 ex 2019/20).

### 2.1 Effects of early‐life stress experience on adult color pattern

The design of the full‐sib split‐brood rearing experiment has been described in detail in Ziegelbecker and Sefc (2021). Wild‐caught adults of *T*. “Ikola” were purchased from an ornamental fish trader and breeding pairs represented both similar and dissimilar parental bar widths (supplementary Figure S1). Some individuals bred more than once, but never with the same partner. *Tropheus* are maternal mouthbrooders, and offspring were mouthbred by their mothers for 2 weeks before being removed from the buccal cavity. The full sib groups, which encompassed 4–15 individuals, were then first transferred to aerated breeding boxes (17 × 12.5 × 13.5 cm L × W × H) and later to tanks (60 × 30 × 30 L × W × H) once they had absorbed their yolk sacs at the age of 20–27 days. At the age of 11–12 weeks, half of the individuals of each full sib group were submitted to an intermittent feeding treatment (the IF group), while the other half served as control group. Juveniles in the control group were fed algal flake food ad libitum on 5 days a week from Monday to Friday, and juveniles in the IF regime were fed ad libitum as well, but only 3 days a week (Monday, Wednesday, Friday). During the 20‐week‐long treatment period, both IF and control fish were housed individually in 15 × 15 × 30 cm compartments that were separated from each other by aluminum micromesh (grid size 1 × 1 mm) within 60 × 30 × 30 cm tanks (8 compartments per tank; with either all IF or all control fish in a tank). Chemical, acoustic, and visual interaction was possible across compartments.

Growth of fish was monitored during the treatment period and was significantly reduced in the IF group (Ziegelbecker & Sefc, 2021). At the end of the treatment period, the fish were approximately 8 months old and most of them started to develop the adult color pattern. From there on, all fish received the same ad libitum food rations five times a week, and were photographed every 4 weeks for measurements of bar width over a period of 2 years. Yellow bars only started to develop at the beginning of the monitoring series. For most fish, it took several months until the bar pattern was fully developed and bar width could be measured (median fish age at first measurement = 15.8 months). For photography, fish were placed in a small aquarium with black background illuminated from the front by a LED lamp, and gently held (by hand) parallel to the front glass of the aquarium next to a ruler (Figure 1). Bar width was measured from the photographs using imageJ (version 1.50i, Schneider et al., 2012). At the same occasion, standard length (SL) was measured by hand with a ruler. Relative bar width (RBW) was then calculated as bar width divided by standard length. RBW measurements could be obtained from 73 progeny (13 females and 22 males in the IF group and 11 females and 27 males in the control group), representing 12 full sib families bred by seven dams and nine sires (Figure S2). Unbalanced sex ratios within the treatment groups are due to the fact that sex of the progeny could not be determined before maturation. Bar width measurements of individual fish were taken over a period of 27–588 days (median = 291 days) and RBW values were highly repeatable across time (442 measurements of 105 fish; repeatability *R* = 0.86, *p* < .001). The RBW of the last measurement per fish was used in further analyses.

To test for an effect of treatment on adult color pattern, we fitted a linear mixed model (LMM) with relative bar width of individuals (“RBW”) as the response variable and “treatment” (intermittent feeding/control) as a fixed factor. We included “brood identity” as random intercept to account for nonindependency among full‐sibs. We used an analogous model to test whether treatment had an effect on the age, at which the fish developed the adult color pattern. For age at color pattern development, we used the age of the fish when it was for the first time possible to measure their bar width in the course of the monthly imaging sessions. The analyses were conducted in R version 3.6.1. (R Core Team, 2019). Within‐individual repeatability of bar width measurements was tested using the rptGaussian function of the package rptR (Stoffel et al., 2017). LMMs were fitted with the packages “lme4” (Bates et al., 2015) and “lmerTest” (Kuznetsova et al., 2017). Compliance with model assumptions was confirmed using the check_model function of the package “performance” (Lüdecke et al., 2021).

### 2.2 Immediate effects of stress exposure on color pattern in adult fish

In this experiment, we exposed adult *T*. “Ikola” to a combination of stressors, consisting of complete food deprivation, poorly structured tank environment, lack of social contact, and daily disturbance, for a period of 18 days. This period proved long enough to produce a clear deterioration of body condition. The 49 wild‐caught *T*. “Ikola” used in this experiment (26 females, 23 males) were different individuals than those used in the rearing experiments, and we included only individuals that were in apparently good condition. Prior to the experiment, fish were held in a group. At the start of the experiment, fish were weighed on a laboratory scale to the nearest 10 mg and bar width was measured as described above. Fish were then individually housed in thoroughly cleaned tanks of 60 × 35 × 30 cm (L × W × D) containing no structure except fine aquarium gravel and an aquarium filter and opaque blends between neighboring tanks. From then on fish were not fed for 18 days. Starting on the second day of the treatment, fish were chased with an aquarium net, to which we had attached yellow and red ribbons, for 60 seconds twice a day (with 4 hours break in‐between) during weekdays. We applied the net‐chasing treatment in randomized order to the test fish in order to avoid predictability of the stressor. Fish were left undisturbed for 2 days on the weekend. Besides food deprivation (Pascual et al., 2003), chasing with aquarium nets induces measurable stress in fish (e.g., Barcellos et al., 2011; Xu et al., 2022). After 18 days, the experiment was terminated and fish were photographed and weighed again. We slowly re‐fed individuals in their separate tanks before they were returned to same‐sex group tanks. We used paired t‐tests to test for changes in weight and bar width between the post‐ and pre‐treatment measurements.

### 2.3 Parent‐offspring regression and estimates of variance components

To test for correlations between parent and offspring RBW and to estimate components of RBW variance, we used the bar width data of the 73 progeny fish and their parents from the split‐brood rearing experiment described above, as well as 40 additional progeny (15 females, 25 males) produced in our lab and reared in groups of similar aged fish with group sizes ranging from 3 to 16 individuals. Tank sizes were 60 × 35 × 60 cm (L × W × D) for groups of 10 or more juveniles and 60 × 35 × 30 cm for smaller groups. We refer to the three rearing conditions as IF, control, and standard. The 113 juveniles included in this analysis originated from 13 different dams and 12 different sires and represented 20 full‐sib families, among which 13 full‐sib families were paternal half‐sibs to one or more other full‐sib families, 10 full‐sib families were maternal half‐sibs with one or more other full‐sib families, and three full‐sib families did not share a parent with any other sib group (Figure S2). Parents were shared among all three rearing conditions.

Mid‐parent‐mid‐offspring regression models were calculated using average RBW within broods as independent variable and mid‐parent RBW as fixed factors (*n* = 20 broods) using the “lm” function in R. Then, we used the R packages “lme4” (Bates et al., 2015) and “lmerTest” (Kuznetsova et al., 2017) to fit linear mixed models (LMM) with individual offspring RBW as response variable (*n* = 113 offspring), including dam RBW and sire RBW as two separate predictors and brood identity as random factor. Initially, rearing conditions (fixed effect factor) and offspring sex (interaction terms with sire and dam RBW and main effect term after dropping the interaction term) were included in the model, but were nonsignificant and dropped from the final model. Compliance with model assumptions was confirmed using the check_model function of the package “performance” (Lüdecke et al., 2021).

Next, we used the Bayesian approach implemented in the R package MCMCglmm to partition genetic and environmental sources of variance in RBW (Hadfield, 2010). We fitted animal models as generalized linear mixed models with a Gaussian error distribution and RBW as the response variable. Pedigree information listed dams and sires of the laboratory‐reared progeny. We had no information about kinship among the wild‐caught parents, but genetic analyses performed by Koch et al. (2008) suggest that due to large population sizes of *T*. “Ikola,” a random sample of wild‐caught adults is unlikely to include close relatives. In the initial model, we included “sex” of individuals and “rearing condition” (three levels: IF, control, standard; see above) as fixed factors. A comparison of DIC values indicated that the fixed factors did not contribute to model fit, and they were therefore dropped from the model. We then explored alternative random effects structures. The full random effect model included offspring identity (“animal”), dam identity (“dam”), and brood identity (“brood”) nested in “dam.” The term “animal” estimates the additive genetic effect; “dam” estimates any shared genetic or environmental maternal effects experienced by full sibs and maternal half sibs; and “brood” was nested in “dam” because several dams produced more than one brood. Including “brood” accounts for shared conditions experienced by full sibs during mouthbrooding, such as the immunological environment of the dam's buccal cavity (Keller et al., 2018), and for brood‐size dependent variation in the social environment during mouthbrooding and prior to the split‐brood treatment.

We ran the same models using a reduced dataset, from which we excluded those three full‐sib groups, which were not related to any other full‐sib family (broods number 14, 26, and 27), in order to reduce the degree to which environmental effects were confounded with the pedigree structure.

We used standard weakly informative priors (*V* = 1, nu = 0.002; as proposed by de Villemereuil, 2012) on residual and random effects variances. A prior sensitivity analysis was carried out on the full random effects model with nu ranging from 0.0005 to 0.05 (Figure S3). Estimates of additive genetic variance were rather insensitive to variation of nu, except for the highest values of nu (Figure S3). Increasing values of nu increased the estimates of for effects of dam and brood. Overall, priors similar to the standard setting (up to nu = 0.005) yielded similar results for all variance components. For each model, we ran 20 million iterations and sampled every 500 times (thinning interval) after a burn‐in of 100,000 iterations. These settings resulted in low autocorrelation (absolute values below 0.005) and high effective sample sizes (>39,000) for the estimated variance components.

In order to examine whether the quantity and quality (e.g., the pedigree information) of our data were sufficient to distinguish between confounded additive genetic and environmental sources of variation, we simulated 100 datasets of offspring RBW data based on the pedigree and parental phenotypes of the empirical dataset. The simulation used additive genetic effects and random noise as the only sources of variance of RBW. For each progeny, the simulated RBW was calculated as mid‐parent RBW plus a random number drawn from a normal distribution with a mean of zero and a standard deviation of 0.03. The standard deviation was chosen to yield a similar distribution of offspring RBW relative to mid‐parent RBW as observed in the empirical data. In the empirical data, mid‐parent RBW explained 36% of the total variance in progeny RBW (linear model not corrected for nonindependence among related offspring); in the simulated datasets, mid‐parent RBW explained 33% of the total variance in progeny RBW (median of 100 simulated datasets).

## 3 RESULTS

### 3.1 Food restriction during juvenile development had no effect on width of adult yellow bar

The average relative bar width of IF‐treated fish was 1.5 percentage points higher than that of control fish (*n* = 12 broods, using mean RBW of IF and control fish within each brood). This difference was not statistically significant (LMM, *n* = 73 fish, est. = 0.009, *t* = 1.43, *p* = .159). There was also no effect of rearing conditions on the age at which the fish developed the adult pattern (LMM, est. = 15.66, *t* = 0.653, *p* = .516).

### 3.2 Adult color pattern did not respond to temporary stress exposure

Fish lost 6.8 ± 1.6 % (1.6 ± 0.4 g; mean ± SD) of their pre‐treatment weight during the experimental stress exposure (paired t‐test for weight difference before and after treatment, *t* = 28.1, *df* = 48, *p* < 2.2 × 10^−16^). Pre‐ and post‐treatment measurements of relative bar width differed by 0–2.3 percentage points (0 to 1.9 mm bar width), with both higher and lower post‐treatment values (means of differences: −0.14 percentage points, or −0.1 mm BW). The change in bar width measurements was not statistically significant (paired t‐test, *t* = 1.1, *df* = 48, *p* = .29), and intra‐individual differences most likely represent measurement noise.

### 3.3 Color pattern of progeny co‐varied with parental phenotypes

**FIGURE 2:**
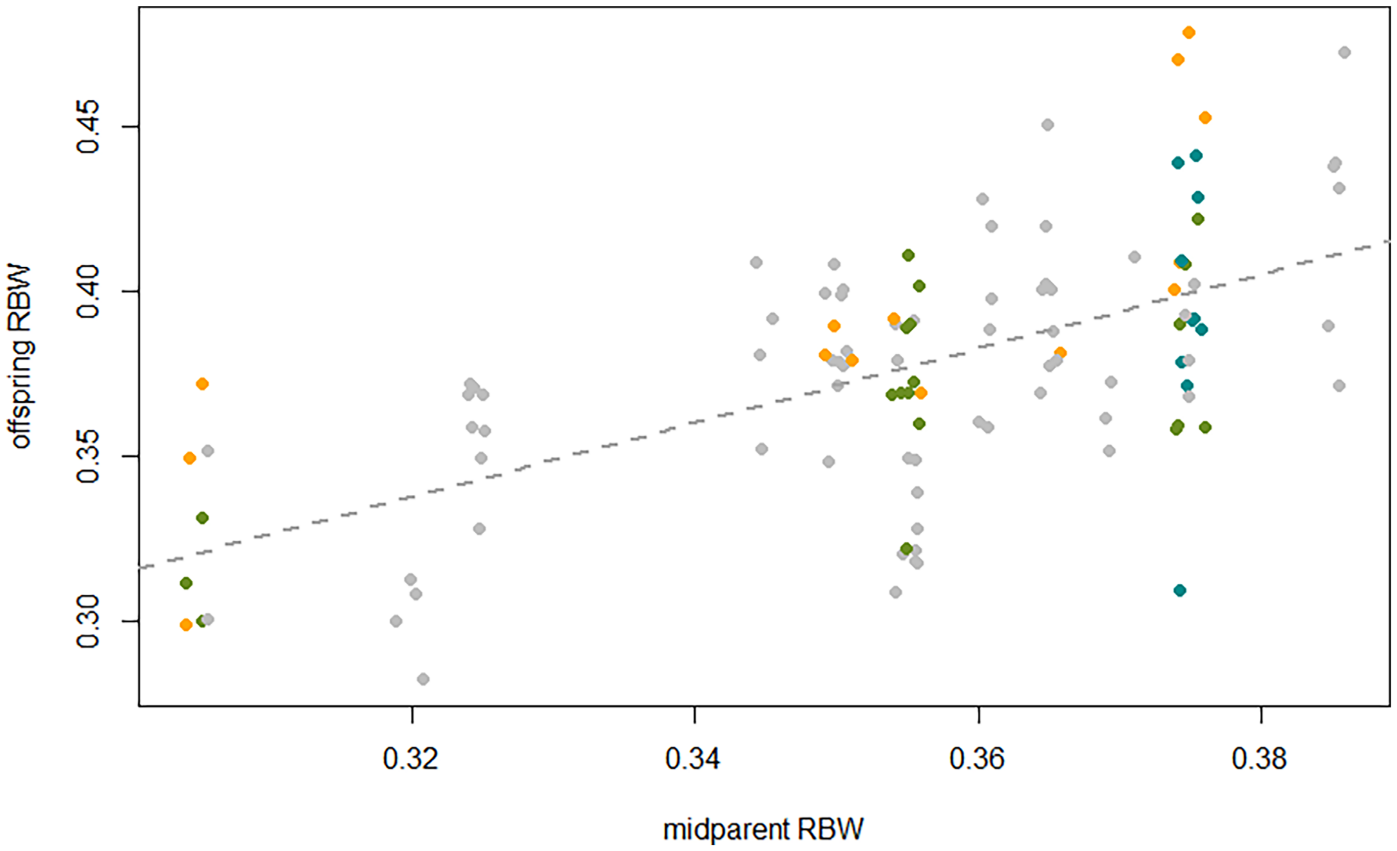
Parent‐offspring correlation of relative bar width (RBW). Individual offspring RBW values are plotted against the mean RBW of their parents. Dots are drawn with small jitter, as some parent pairs had identical mean RBW. In these cases, offspring of different parent pairs are distinguished by different dot colors. The broken line illustrates the mid‐parent‐mid‐offspring regression equation.

This analysis used data from 113 lab‐bred adult fish with known parent phenotypes, including the 73 fish raised in the split‐brood experiment as well as 40 fish raised under standard conditions. Offspring RBW was positively correlated to mid‐parent RBW (Figure 2). Mid‐parent RBW explained 67% of the variance among the mean RBW values of the full‐sib groups (linear model, est. = 1.13, *t* = 6.33, *p* = 5.75 × 10^−6^, *n* = 20 broods). Modeling maternal and paternal RBW as two separate predictors of offspring RBW (*n* = 113 offspring), their effect sizes were similar to each other and both were statistically significant (LMM, est_dam_ = 0.60, *t* = 3.7, *p* = .002, est_sire_ = 0.52, *t* = 2.3, *p* = .034).

### 3.4 Estimations of variance components in animal models

This analysis used the same offspring data as the parent–offspring regression analysis (*n* = 113 offspring). The full model included sex of fish and rearing condition as fixed factors, and three random factors: “animal” (additive genetic effects), as well as shared early environment represented by “dam” (maternal effects), and “brood” nested in “dam” (environment experienced during mouthbrooding and full‐sib rearing). Neither of the two fixed factors had a significant effect on RBW, and DIC decreased slightly when the fixed factors were dropped from the model (DIC of the full model: −500.41; DIC after fixed were dropped: −501.98). A step‐wise elimination of the random effects terms further decreased the DIC values and identified the simplest model, including only additive genetic effects, as the best model (Table 1). However, each of the three different random effects models explained a similar proportion of total variance (79–85%), which was divided approximately equally among the included random terms (Table 1).

**TABLE 1:** Animal model variance ratio estimates.

	Random effects: Animal, dam, brood	Random effects: Animal, dam	Random effects: Animal
Empirical data			
DIC	−501.978 (−425.78)	−506.74 (−429.33)	−520.27 (−435.10)
Total variance explained	0.852 (0.845)	0.833 (0.799)	0.793 (0.724)
Additive genetic	0.193, 0.057–0.414 (0.196, 0.060–0.450)	0.332, 0.116–0.602 (0.357, 0.134–0.639)	0.793, 0.574–0.910 (0.724, 0.424–0.880)
Dam	0.289, 0.105–0.629 (0.264, 0.090–0.604)	0.406, 0.198–0.731 (0.328, 0.133–0.665)	–
Brood	0.188, 0.063–0.468 (0.185, 0.061–0.452)	–	–
Simulated data			
DIC	−477	−481	−488
Total variance explained	0.79	0.75	0.67
Additive genetic	0.21	0.30	0.67
Dam	0.24	0.33	–
Brood	0.16	–	–

*Note*: Statistical support by DIC, proportion of total variance in RBW explained by the model, and proportions of total RBW variance assigned to additive genetic effects, maternal effects, and brood effects. For the empirical data, model estimates are reported as posterior modes and 95% HPD intervals. Variance ratio estimates for the reduced empirical dataset, from which we excluded those three full‐sib groups, which were not related to any other full‐sib family, are given in parentheses. For the simulated data, we report the median values of the outcomes (DIC, proportion of total RBW variance explained by the model, and posterior modes of proportions of total RBW variance assigned to each effect) across the 100 simulated datasets.

To reduce the extent, to which the sources of variation were confounded in our dataset, we removed three full‐sib families, which were not related to any other full‐sib family, from the dataset. The results remained similar, in that the simplest model, including only additive genetic effects, had the lowest DIC score. Again, the proportions of total variance explained by each of the three alternative random‐effects models were distributed among all included variance components (Table 1). Hence, with both datasets, information‐criterion‐based model selection supported the model that estimated a high heritability of RBW (0.79 or 0.72, respectively; Table 1), but considerable proportions of variance were also attributed to maternal and environmental effects when the corresponding terms were included.

We wondered whether the sample size and pedigree structure of our data were insufficient to accurately disentangle additive genetic and environmental effects. To investigate whether the absence of maternal and environmental effects would be correctly recognized in such a dataset, we used the pedigree structure and the parent phenotypes of our empirical data (Figure S2) and simulated progeny RBW as being determined by additive genetic effects with a certain amount of random noise (see Methods). Using the simulated data, we modeled the same three random effects structures as with the empirical data. Compared to the model including only the additive genetic effects term, adding the terms “dam” and “brood” increased the proportion of explained variance from 67% to 79% (medians of 100 simulations) and decreased estimated additive genetic effects from 0.67 to 0.21 (medians of posterior modes across 100 simulations), with maternal and environmental effects accounting for the remaining proportions of explained variance (Table 1). DIC values decreased when the (nonsimulated) effects of dam and brood were excluded. Hence, the models matching the simulated situation were recognized as the statistically best models, but nonsimulated (i.e., zero) effects of dam and brood were not estimated correctly.

## 4 DISCUSSION

The wide yellow bar of *T*. “Ikola” is linked to competitive success and territorial behavior (Ziegelbecker et al., 2018, 2021) and is therefore a likely candidate for a social signal. In a very straightforward way, the width of the yellow bar might serve as an easy‐to‐read index of body size. Indeed, body size explains 50% to 60% of the variance of absolute bar width (data from this study and from Ziegelbecker et al., 2018). However, the body size‐corrected effects of bar width (i.e., RBW) on social interactions and exploration behavior (Ziegelbecker et al., 2018, 2021) suggested that RBW may carry additional information and for instance reflect variation in physiological condition. Here, we tested for environmental influences on the width of the yellow bar and found that it did not respond to experimentally induced variation in conditions prior and after the formation of the adult color pattern. In both experiments, the treatments had measurable effects on other traits. The stress level administered to the adult *T*. “Ikola” simulated a severe deterioration of living conditions, such as might be experienced in nature upon the loss of the territory. The applied stressors—food deprivation and chasing—have previously been demonstrated to induce responses of stress biomarkers in fish (Barcellos et al., 2011; Pascual et al., 2003; Xu et al., 2022). In our experiment, the fish responded with considerable weight loss and hence a decrease in their condition factor. Photographs taken for measurements of bar width before and after the stress treatment also suggested that coloration became paler during the treatment period. In contrast, the treatment induced neither morphological (Leclercq et al., 2010) nor physiological changes (Sköld et al., 2013) of the width of the yellow bar. Relative bar width had also remained constant in adult *T*. “Ikola” held in normal, benign lab conditions and monitored over a period of up to 600 days (Ziegelbecker et al., 2018). Together, these results suggest that once developed, the adult color pattern of *T*. “Ikola” is stable over time as well as in the face of changing conditions. This contrasts with environmental responses of adult color patterns in other fish species. For instance, in the cichlid fish *Pundamilia nyererei*, the red area expressed by adults responded rapidly to social stimulation by rivals and increased in size within 4 days of exposure (Dijkstra et al., 2007). Intriguingly, food‐deprived three‐spined stickleback males developed *larger* areas of nuptial red coloration than control fish, suggesting dishonest signaling driven by residual reproductive value (Candolin, 1999).

The food shortage experienced by juveniles in the rearing experiment simulated mild but protracted stress during development that might, under natural conditions, result from foraging competition or predation pressure during the juvenile phase. In the experiment, the juveniles reduced their growth rates at the start of the IF treatment but maintained constant size‐to‐weight ratios (Ziegelbecker & Sefc, 2021). The growth‐retarded IF fish caught up in body size after the treatment ended and displayed no deficits in their competitive ability compared to the control group (Ziegelbecker & Sefc, 2021). The present data show that the adult color pattern remained equally unaffected by the nutritional deprivations experienced as juveniles. While lingering effects on other phenotypic traits cannot be ruled out, the fish appeared resilient to adverse early‐life conditions with respect to the measured variables (adult body size, color pattern, and competitive ability). We caution that this result refers to the stress level differences between treatment and control groups applied in our experiment, which may have been sufficient to modify growth rates but not adult trait expression. Nonetheless, numerous examples attest to the effects of early‐life experiences on adult phenotypes (Royle et al., 2015), including the size of color patches displayed on the body (Ruell et al., 2013; Walker et al., 2013; Wilson et al., 2019). Remarkably, even though the size of the orange area on the body of guppies has been shown to be highly heritable in several studies (Brooks & Endler, 2001; Houde, 1992; Sato & Kawata, 2020), the trait responded rapidly to food restrictions administered to adult male guppies (Cattelan et al., 2020; Devigili et al., 2013), as well as to the exposure to predator cues and to variation in food supply during rearing (Ruell et al., 2013). In contrast, similar to our observation, fire salamander larvae exposed to different nutritional conditions did not differ in the area of yellow body color after metamorphosis, although individuals reared under the rich dietary regime were more brightly colored (Barzaghi et al., 2022).

While stress exposure in our experiments did not contribute to variation in bar width, our data suggested that bar width in *T*. “Ikola” is influenced by genetic and possibly also nongenetic parental effects. Mid‐parent‐mid‐offspring regression models revealed a significant correlation of RBW between parents and their offspring. The effect size of the mid‐parent RBW on the mean RBW of their offspring was close to 1, suggesting that on average, an increase in mid‐parent RBW by 1% corresponds to a 1% increase in progeny RBW. The parental effect was divided almost equally between the two parents when dam and sire RBW were modeled as separate predictors, suggesting comparable maternal and paternal contributions to offspring RBW. We then used animal models to separate additive genetic from other sources of variation. In our breeding experiment, pedigree structure was confounded with early‐life environment, as members of a brood shared the same immunological and social environment during mouthbrooding and subsequent full‐sib group housing (Keller et al., 2018), and maternal half‐sibs shared maternal influences. Shared environmental conditions can introduce a bias in the estimates of additive genetic effects, and the inclusion of rearing environment and maternal effects in animal models is recommended to safeguard against the overestimation of heritability (Kruuk & Hadfield, 2007; Thomson et al., 2018; Wilson et al., 2010). In the present case, the inclusion of dam and brood effects was not supported by the outcome of DIC‐based model selection, which identified the additive‐effects model as the statistically best model. Nonetheless, considerable proportions of variance were attributed to other sources (dam, brood) when these were included. We note that pedigree depth and sample size in our dataset may limit our ability to statistically separate environmental and nonadditive genetic from additive genetic effects (Kruuk & Hadfield, 2007; Wilson et al., 2010). The simulations suggested that the observed outcomes in terms of DIC and variance partitioning in the alternative models were compatible with additive genetic effects as the main source of variation in bar width. However, this does not prove that dam and brood effects are indeed absent. A comparable example to the present case has been hypothetically drafted by Wilson et al. (2010). Following their advice to present and consider the competing models, we cautiously conclude that our data revealed an association between the pedigree and the phenotype that is consistent with heritability of the trait, while additional effects of shared environments and shared mothers cannot be excluded. We note, however, that an effect of shared egg‐stage and fry‐stage environment on color pattern (factor “brood” in the animal model) would contrast with the insensitivity of the adult color pattern to the experimental manipulation of rearing environments. In this light, the existence of “brood” effects would indicate that the experimental feeding treatment missed the window of responsiveness. Alternatively, environmental effects on bar width may differ between groups of relatives (i.e., in different genetic backgrounds), such that environmentally induced variation among sibling groups, which would be recognized as maternal and brood effects on top of additive genetic effects, would not necessarily be correlated with environmental gradients.

Despite some degree of uncertainty in the partitioning of variance components, we consider it relevant that a large proportion of the total variance in bar width was explained by the random effects included in the alternative models. This suggests that variance in bar width largely reflects effects of provenance, either mediated by genetic or environmental factors, rather than random outcomes of chromatophore migration and autonomous pigment cell interactions or environmental influences later in life. Consistent with a strong heritable or early‐life component of bar width variation, experimentally induced stress of juveniles and adults had no measurable effect on the color pattern. Based on these findings, bar width of *T*. “Ikola” might provide information on genetic quality or on the quality of very early life (egg, embryo, or fry) environment to competitors or potential mating partners, while current condition might perhaps be signaled through variation in color intensity.

## AUTHOR CONTRIBUTIONS

**Angelika Ziegelbecker:** Conceptualization (equal); data curation (equal); formal analysis (equal); investigation (equal); methodology (equal); project administration (equal); writing – original draft (equal); writing – review and editing (equal). **Kristina M. Sefc:** Conceptualization (equal); data curation (equal); formal analysis (equal); funding acquisition (lead); methodology (equal); project administration (lead); supervision (lead); writing – original draft (equal); writing – review and editing (equal).

## CONFLICT OF INTEREST STATEMENT

The authors have no conflicts of interest.

## Supporting information


Figure S1.

Data S1.

## Data Availability

Data used in this manuscript are provided as supplementary material.
