# Supplementary material for: Family resemblance in color‐patch size is not affected by stress experience in a cichlid fish
Source: Ecol Evol. 2024 Jul 21;14(7):e70009. doi: 10.1002/ece3.70009 (PMC11260441; doi:10.1002/ece3.70009)
Supplement: Supplementary file 1 — Figure S1. [file ECE3-14-e70009-s002.docx]

**Family resemblance in color-patch size is not affected by stress experience in a cichlid fish**

Angelika Ziegelbecker, Kristina M. Sefc


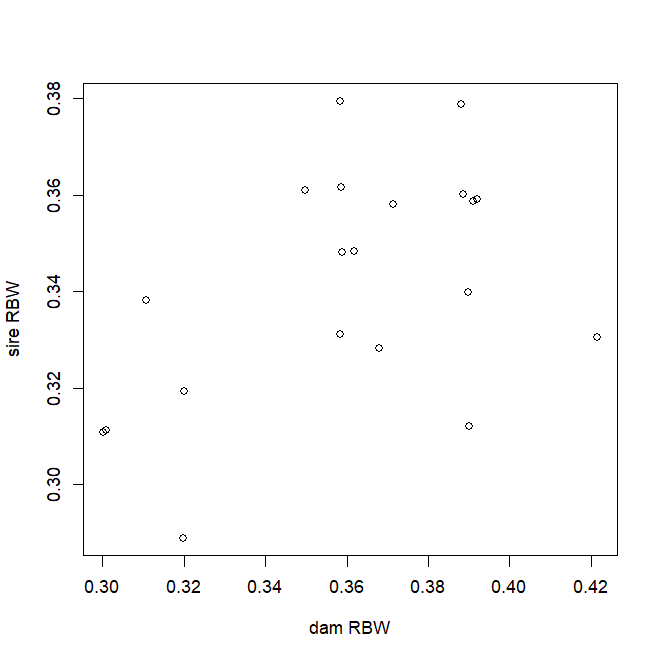


Supplementary Figure 1: Relative bar width (RBW) of sires and dams producing the 20 broods of offspring used in this study. Dots are drawn with small jitter as some breeding pairs overlap in their RBW values.


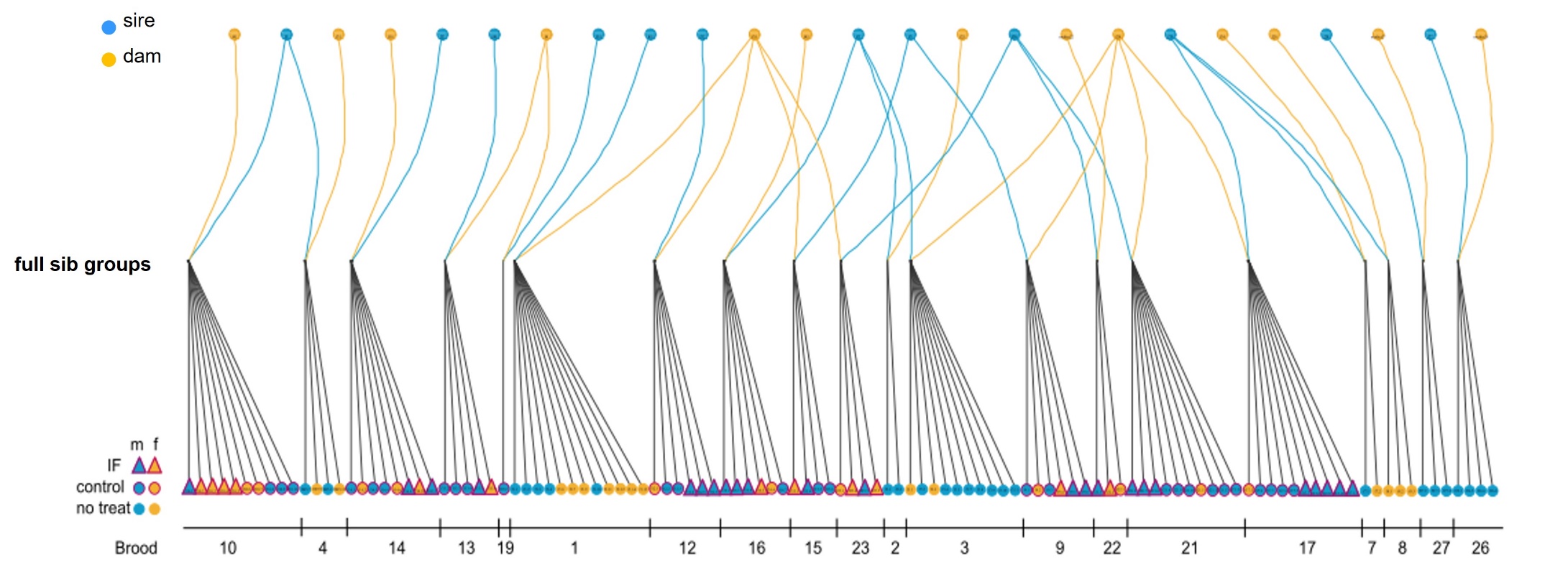


Supplementary Figure 2: Pedigree of the 113 offspring used in this study. Dots in the upper row represent breeding dams (orange) and sires (blue), connected by lines to their full-sib offspring groups. Offspring symbols represent rearing conditions: intermittent feeding treatment in the split-brood experiment (‘IF’), control treatment in the split-brood experiment (‘control’), and group rearing under standard lab conditions (‘no treat’). Brood ID is given on the bottom line.

Supplementary Figure 3: Prior sensitivity analysis. Estimates (on y-axis) for additive genetic variance (heritability), maternal effects (dam) and brood effects obtained in animal models with nu ranging from 0.0005 to 0.05 (on x-axis). The table below the x-axis reports the estimated variance components and the total variance explained by the model for each value of nu.
